# Advances in antitumour therapy with oncolytic herpes simplex virus combinations

**DOI:** 10.1007/s12672-024-01165-z

**Published:** 2024-07-24

**Authors:** Xuejiao Qi

**Affiliations:** College of Basic Medical Sciences, China Three Gorges University, Yichang, Hubei, China

**Keywords:** oHSV, Combination therapy, Tumour

## Abstract

Oncolytic Virus (OVs) is an emerging approach to tumour immunity that allows the use of natural or genetically modified viruses to specifically infect and lyse tumour cells without damaging normal cells. Oncolytic herpes simplex virus (oHSV) is one of the more widely researched and applied OVs in the field of oncology, which can directly kill tumour cells to promote anti-tumour immune responses. oHSV is one of the few viruses with good antiviral drugs, so oHSV is also more clinically safe. In recent years, in addition to monotherapy of oHSV in tumours, more and more studies have been devoted to exploring the anti-tumour effects of oHSV in combination with other therapeutic approaches. In this article we describe the progress of oHSV combination therapy against tumours in the nervous system, digestive system, reproductive system and other systems.

## Introduction

In recent years, epidemiological surveys have shown that the global incidence of tumours is increasing year by year, and tumours are not only one of the major diseases that threaten human life and health, but also pose a great threat to the economy of human society. At present, the main treatment modalities for tumours in clinical practice include surgery, radiotherapy chemotherapy and targeted therapy. However, there are still many problems with these treatments, and it is urgent to find effective emerging anti-tumour methods. Oncolytic Virus (OVs) is a new type of tumour immunotherapy, which can selectively infect tumour cells, self-replicate in tumour cells and evade antiviral immunity to achieve anti-tumour effects [1]. Herpes simplex virus (HSV) is a neurotropic double-stranded DNA enveloped virus of the subfamily α herpesviruses, which can be classified into two serotypes according to its antigenicity, Herpes simplex type I (HSV-1) and Herpes simplex type II (HSV-2). The genome length of HSV-1 is 125-240 kb and that of HSV-2 is around 154 kb. HSV can be genetically modified to transform into oncolytic herpes simplex virus (oHSV), which is of widespread interest in oncology therapy. Compared with other OVs, oHSV has the following advantages: (1) large viral genome capacity, which can be easily modified to carry multiple exogenous therapeutic genes [2]; (2) broad spectrum of infection, capable of infecting most of the tumour cells; (3) high infection efficiency, capable of rapid replication, short tumour lysis time, and strong infectious activity; (4) low impact on normal cells, which is safe and weakly pathogenic; and (5) it has been shown that the combination of oHSV therapy produces a comprehensive response to improve its anti-tumour efficacy compared with other anti-tumour therapies [3]. This article focuses on current progress in combining oHSV and other types of drugs against tumours.

## Tumours of the nervous system

### Glioma

Glioma, which originates from glial cell lesions in the brain and spinal cord, is the most common intracranial malignant tumour, accounting for 27% of all central nervous system tumours. It is characterised by high morbidity, recurrence and mortality rates and low cure rates, and is considered one of the most difficult tumours to treat in the field of neurosurgery. The treatment of glioma is based on surgical resection, combined with a combination of radiotherapy, chemotherapy and targeted therapy, but glioma is difficult to cure, and many researchers and scholars are looking for new treatments for glioma, especially combination therapy. The following summarises the current oHSV-related combination therapy for glioma.

Bevacizumab is the first targeted anti-tumour angiogenesis drug approved for marketing, which exerts anti-tumour effects by specifically targeting vascular endothelial growth factor (VEGF), and has achieved promising results in the treatment of a wide range of malignancies. It has been shown that anti-VEGF therapy induces glioma invasion, and the combination with integrin inhibitors exerts a significant anti-invasive effect [4]. Yusuke Tomita et al. produced a vasopressor-expressing lysosomal herpes simplex virus-1 (RAMBO) to study its effect on bevacizumab-induced glioma invasion [5], who speculated that the RAMBO-expressing vasopressor might antagonise the integrin-related pathway induced by bevacizumab, thereby reducing glioma invasion. In in vitro assays, migration and invasion assay of RAMBO-infected cells were performed, and the results showed that bevacizumab significantly increased the proportion of glioma cells invaded. Whereas combination therapy with RAMBO and bevacizumab significantly inhibited bevacizumab-induced glioma cell migration and invasion, analysis of the anti-tumour effects of the combination in xenograft mice, it was found that the median survival of combination-treated mice was significantly prolonged compared to either monotherapy. Subsequent IHC staining of postmortem glioma mice using anti-human leukocyte antigen, followed by assessment of the depth of glioma infiltration by the distance between the edge of the tumour mass and the zone of invasion, revealed that the combination treatment significantly reduced the depth of glioma infiltration induced by bevacizumab.

The MAPK signalling pathway plays an important role in cell value-adding, differentiation, apoptosis, etc. Abnormal activation of the MAPK signalling pathway (RAS-RAF-MEK-ERK signalling pathway) is more common in gliomas, and targeted drugs against the MAPK pathway play an important role in a variety of tumour treatments, however, monotherapy is associated with the problem of tumour resistance.The MEK kinase inhibitor (MEKi) trametinib is one of these targeted agents, but its low penetration in the central nervous system limits its use in brain tumours [6]. Ji Young Yoo et al. combined trametinib with oHSV for the treatment of glioma. In an experiment on the effect of oHSV treatment on the in vivo penetration of trametinib into brain tumours, treatment of mice bearing intracranial U87ΔEGFR human gliomas showed a significant enhancement of targeted anti-tumour effects with the addition of oHSV, which increased the percentage of brain-plasma exposure of trametinib in the tumours by nearly 600% compared to those treated with trametinib alone; also in the experiment, it was found that trametinib treatment reduced macrophage-induced TNFα secretion, but the immunity of mice was not reduced, and the use of combination therapy enhanced T-cell-mediated anti-tumour efficacy. In analysing the Kaplan–Meier survival curves of mice with intracranial GL261-N4 gliomas, it was found that combination therapy significantly improved survival, and that this survival benefit was due to the long-term protection of CD4 + and CD8 + T cells [7].

Copper is an important cofactor for certain angiogenic factors and is required for the secretion of angiogenic factors by tumour cells [8]. Elevated serum and tissue copper levels have been associated with cancer development [9], targeting copper is also a new strategy for cancer treatment. ATN-224 is not only a novel copper chelator but also an anti-angiogenic agent [10]. Ji Young Yoo et al. tested the anti-tumour efficacy of the combination of ATN-224 and oHSV for the first time. In in vivo experiments in mice bearing intracranial gliomas, the combination-treated mice showed not only significant inhibition of tumour growth, but also significant improvement in survival and significant improvement in anti-tumour efficacy as compared to monotherapy. Further experimental analyses revealed that in addition to reducing tumour angiogenesis, this combination therapy, in which ATN-224 also rescued copper-mediated inhibition of oHSV replication and cytotoxicity, enhanced the anti-tumour effect. To further investigate the correlation between this combination therapy, they injected a single dose of oHSV via the tail vein into tumor-bearing mice treated with either ATN-224 or phosphate-buffered saline (PBS), and collected mouse serum 20 min later to assess the number of viral particles, and found that the number of viral particles present in the serum of the ATN-224-treated mice increased dramatically compared to that of the PBS-treated mice. It is evident that ATN-224 treatment improved the serum stability of oHSV in vivo and enhanced the antitumour efficacy and systemic delivery of oHSV [11].

Meta-iodobenzylguanidine (MIBG) is a norepinephrine analogue capable of active uptake by norepinephrine transporter proteins (NATs).The 131I-labelled form of MIBG is clinically used as a tumour-targeting radiopharmaceutical for the diagnosis and treatment of adrenergic tumours, but is restricted to a small number of neural crest-derived tumours [12]. Ji M. Quigg et al. designed a NAT-expressing ICP5.1716 null mutant (HSV-1) of HSV34.5, named HSV1716/NAT, and introduced the NAT gene into glioma cells using plasmid-mediated transfection to explore the feasibility of a combined therapeutic approach for glioma treatment with HSV1716/NAT and [131I]MIBG in in vitro experiments [13]. It was demonstrated that the NAT gene in cultured glioma cells introduced by HSV1716/NAT began to be expressed 1 h after viral infection and was able to actively uptake [131I]MIBG. The combination of HSV1716/NAT and [131I]MIBG reflected dose- and time-dependence leading to significantly enhanced cytotoxicity compared to either drug alone. The combination of HSV treatment with targeted radiotherapy has the potential to effectively kill tumour cells, so in vivo experiments were subsequently evaluated by the relevant groups. Annette Sorensen et al. in tumour xenografts that do not express NAT, intratumoural or intravenous injections of HSV1716/NAT induced the active cellular uptake of of 131I-MIBG [14]. The results demonstrated that the combination treatment resulted in reduced tumour growth and improved survival relative to injection of either drug alone, further confirming the usefulness of HSV1716/NAT for gene therapy and targeted radionuclide therapy performed on cancers.

## Glioblastoma

According to the 2021 World Health Organization Classification of Tumours of the Central Nervous System, it uses a new approach to differentiate between gliomas, glial neuronal tumours and neuronal neoplasms and divides them into six categories, among which adult-type diffuse gliomas include glioblastoma. It has an incidence of approximately 3 to 8 per 100,000 people per year, and although the prevalence is relatively low, patient survival is relatively short because of its rapid growth rate. The clinical manifestations of glioblastomas are mainly related to the tumour-occupying effect and its impact on adjacent brain functions, which mainly include headaches, epilepsy and mood or personality changes. The current standard treatment for glioblastomas is mainly surgical treatment, supplemented by postoperative simultaneous radiotherapy and adjuvant chemotherapy, but the prognosis of patients is still poor, with a one-year survival rate of 40.6% and a five-year survival rate of only 5.6% [15]. Currently, the combination of multi-target and multiplex therapies is a research hotspot in the diagnosis and treatment of glioblastomas, and the following highlights summarise the oHSV-related combination therapy for glioblastomas.

Rui Ma and his team designed an oHSV called OV-IL15C [16].They used OV-IL15C with frozen EGFR-CAR-NK cells to study them in vitro and multiple glioblastomas mouse models for monotherapy and combination therapy effects. They first tested the effect of monotherapy, survival was significantly prolonged in mice treated with OV-IL15C plus human CD8 T cells compared to control groups, but the same anti-tumour effect was not obtained by replacing CD8 T cells with NK cells. In order to improve the anti-tumour activity of NK cells in immunotherapy, they produced an EGFR-CAR-NK cell, and finally obtained anti- glioblastomas effects in both in vivo and in vitro experiments in xenograft models. Afterwards they performed a combination test of OV-IL15C and EGFR-CAR-NK cells. The result was that both in vivo and in vitro experiments combination therapies showed better results compared to monotherapies: tumour load was significantly reduced and survival of mice was significantly prolonged.

IL-12 is a major regulator of anti-tumour immunity, and Dipongkor Saha et al. designed an oHSV expressing IL-12 (IL12-oHSV) and conducted experiments with IL-12 in combination with immune checkpoint inhibitors (ICIs) [17]. glioblastomas stem cell-like cells (GSCs) can increase the chances of forming tumour spheres, expressing stem cell markers and phenotypically replicating the original tumour in vivo through self-renewal, so GSCs are gradually becoming a key target for immunotherapy. Therefore, they conducted experiments using a mouse GSC-derived tumour model, in which they found that not only was single IL12-oHSV or ICIs ineffective in immunosuppression of glioblastomas, but also the dual combination of IL12-oHSV and ICIs was ineffective. They then chose two ICIs that were independent of the immunosuppressive pathway: anti-CTLA-4 and anti-PD-1, and again performed a combination experiment, which finally revealed a significant effect against GSCs. This triple therapy successfully cured 77% of the mice in a representative GSC-derived immunocompetent 005 glioblastomas model and produced immune memory protection in all triple therapy-cured mice. This anti-tumour effect was associated with a significant reduction in tumour cells and an increase in the ratio of T effector/T regulatory cells. In addition, the immune cell depletion/suppression studies they performed showed that CD4, CD8 T cells and macrophages are all required in triple therapy, with CD4 T cells playing a key role [18].

Podoplanin (PDPN) is highly expressed in many solid tumours. High expression of PDPN has been associated with a poorer prognosis in gliomas [19], PDPN is most abundant in glioblastomas and therefore is considered as an important target for CAR-T cell therapy of brain tumours. Lushun Chalise and other researchers produced a Chimeric Antigen (CAR), a cancer-specific monoclonal antibody against human PDPN (CasMab, LpMab-2) [20]. In their study, they conducted in vitro experiments by targeting G47Δ (third-generation tumour lysing recombinant HSV-1) and Lp2-CAR-T cells to glioblastomas cells, and found the combination of the two treatments further reduced the viability of the cancer cells and inhibited their growth even more significantly; the in vivo experiments not only inhibited tumour growth but also prolonged the survival of mice.

Etoposide (VP-16), a semi-synthetic derivative of the natural antibiotic, disulfiram, is a cell cycle-specific agent and a chemotherapeutic agent for glioblastomas, but has dose-limiting toxic myelosuppressive adverse effects during treatment. Tooba A. Cheema et al. used a low-dose etoposide in combination with G47Δ in human GSC xenografts [21]. In vitro experiments showed that the combination of etoposide and oHSV killed glioblastomas cells, including GSCs, more effectively than either drug alone; after cell cycle analysis, both drugs blocked the cell cycle at different phases, with the combination treatment leading mainly to a significant increase in the sub-G1 population and a decrease in the G2/M phase. In vivo experiments in mice revealed that a single intratumoural injection of G47Δ increased survival, but the combination treatment significantly prolonged median survival, and Most importantly this combination therapy showed no side effects.

## Other types of nervous system tumours

### Meningiomas

Meningiomas are a common type of tumour in the central nervous system, malignant (mesenchymal) meningiomas (MM) is a rare and devastated type of it. The treatment for MMs is mainly surgical resection and radiotherapy, with chemotherapy working in only a small proportion of patients. However, patients with meningiomas have a high rate of postoperative recurrence, often ranging from 13 to 40%, which increases with follow-up time [22]. Therefore, it is necessary to develop new therapeutic approaches. Histone deacetylase inhibitors (HDACis) target HDAC and inhibit its action, regulate the acetylation state of histones, and promote the transcription and expression of tumour transcription factors, and now has being developed as anti-tumour agents. HDACis are one of the candidates for the combination therapy of oHSV, the Yoichiro Kawamura et al. hypothesised that the combination of HDACis and G47Δ is an effective treatment against MM, and to test this hypothesis, they performed in vivo and in vitro experiments using human MM cells and xenograft models [23]. The experimental results showed that HDACis enhanced the replication and therapeutic activity of G47Δ in MM xenograft models in vivo, and improved the ability of xenograft growth to resist metastasis. It is evident that this experimental hypothesis may be applied to clinical drug development.

### Malignant peripheral nerve sheath tumor

Malignant peripheral nerve sheath tumor (MPNST) is a group of malignant tumours originating from peripheral nerves, or nerve sheath membrane cells. According to epidemiological surveys, MPNST accounts for approximately 5% to 10% of all soft tissue sarcomas and can be primary or secondary to neurofibromatosis type I (NF1) [24]. Since Ras and Epidermal growth factor receptor (EGFR) signalling pathways play an important role in the pathogenesis of MPNST, these signalling pathways represent potential targets for MPNST treatment [25, 26]. Erlotinib is an EGFR tyrosine kinase inhibitor, and although it has achieved some efficacy in the treatment of MPNST, the effect of combination therapy with other antitumour therapies remains unclear.Yonatan Y Mahller et al. investigated the efficacy of oHSV in combination with erlotinib in the treatment of MPNST [27]. In a human MPNST xenograft mouse model, the anti-tumour activity of oHSV injection was apparently stronger compared to erlotinib alone, while the combination of the two treatments was more potent against tumour cellular appreciation. In addition, both oHSV and erlotinib were found to have anti-angiogenic effects by detecting the expression of pro-angiogenic genes in mice. It is evident that this combined treatment deserves further in-depth study.

## Tumours of the digestive system or of the spleen

The mitogen-activated protein kinase (MAPK) cascade (RAS/RAF/MEK/ERK) is one of the most dysregulated pathways in human cancers, and one common one is colorectal cancer with KRAS and BRAF mutations [28]. Trametinib is an FDA-approved kinase inhibitor for the treatment of tumours with BRAFV600E and V600K mutations. Zhou and his researchers investigated the therapeutic effect of trametinib in combination with oHSV in KRAS and BRAF mutant colorectal cancer, and they found that this combination therapy not only inhibited the expression of START1 mRNA and PKR in cancer cells, but also reduced the phosphorylation in BRAFwt/KRAS mutant tumour cells, which in turn facilitated the replication of oHSV, and enhanced the anti-tumour effect [29].

Controlling immunosuppression and immune resistance in cancer cells enhances the tumourolytic activity of oHSV, and reducing the impact of bone marrow-derived suppressor cells (MDSCs), which have the ability to significantly inhibit immune cell responses in cancer patients, may increase the efficacy of immunotherapy. Gemcitabine (GEM), a cytosine nucleoside analogue chemotherapeutic agent, has been experimentally shown to directly inhibit MDSCs [30]. Shinichi Esaki and his researchers found that the combination of GEM and oHSV in an experimental colorectal tumour model enhanced the anti-tumour effect of oHSV by selectively inhibiting MDSCs, as evidenced by the inhibition of tumour cells and necrosis in splenocytes, as well as an increase in CD4T and CD8T cells in mice model [31].

Canerpaturev (C-REV) is a spontaneous mutant of oHSV, originally isolated from the herpes simplex virus-1 (HSV-1) strain HF as clone 10 (formerly known as HF10), and is a cancer immunotherapeutic drug that combines direct killing of tumour cells with immunomodulation.EGFR plays an important role in the development and function of normal tissues. However, when it is overexpressed or abnormally activated, it can lead to tumour development and progression.EGFR is an important target that has been clearly identified that can be used in the treatment of colorectal cancer, and cetuximab is one of these important targeted agents [32], but its "endurance" in treatment is limited, and its continued use will not only reduce its effectiveness, but also cause side effects. Wu and his researchers investigated the effects of C-REV in combination with cetuximab in human colorectal cancer [33]. It was found that this combination therapy has a strong synergistic anti-tumour effect on tumour cells with high EGFR expression of HT-29, in addition to a significant enhancement of anti-angiogenic effect. With more in-depth studies, the combination therapy of oHSV may become a new strategy for human colorectal cancer treatment.

Gastric cancer is one of the malignant tumours with the highest morbidity and mortality rate in the world, and due to the lack of clear clinical indications, most of the patients are already in advanced stages when they are diagnosed. Advanced gastric cancer with multiple metastases, especially liver and peritoneal metastases are difficult to treat and have a poor prognosis. The anti-angiogenic effect of bevacizumab can be used to treat various types of metastatic cancers.Tomohiro Deguchi et al. investigated the effect of bevacizumab in combination with the oncolytic herpesvirus hrR3 in a human gastric cancer model under experiments with different concentrations of bevacizumab and viral titers [34]. Analysis of the experimental results showed that the combination of bevacizumab and hrR3 significantly inhibited tumour angiogenesis and increased the distribution of intratumourally injected oHSV. This antitumour effect was more pronounced than using either of them, and is not a promising therapeutic strategy for gastric cancer.

Pancreatic cancer is one of the most common malignant tumours in the digestive tract, and it has the title of "King of Cancers" in the neighbourhood of tumours, which is daunting. In recent years, with the rapid development of molecular targeted therapies, the treatment of pancreatic cancer has moved from a single stage of chemotherapy and radiotherapy to more precise and effective targeted immunotherapy, and new discoveries of various drugs and therapies are challenging pancreatic cancer. As of January 2023, there are four targeted drugs approved by the FDA for the treatment of pancreatic cancer, one of which is erlotinib. Erlotinib is primarily used in combination with GEM for the treatment of locally advanced, unresectable, or metastatic pancreatic cancer, and while this combination significantly prolongs overall patient survival, it is accompanied by a significant increase in toxicity [35]. Kazuo Yamamura et al. attempted to combine erlotinib with HF10 for the treatment of pancreatic cancer [36]. In an in vitro experiment in a human pancreatic cancer xenograft model, the combination of erlotinib and HF10 was found to produce higher cytotoxicity on pancreatic cancer cells than either drug alone. In vivo experiments allow the researchers to further asses the effect of combination. However, the combination treatment was clearly more effective in inhibiting tumour growth when compared to monotherapy. Further evaluation revealed that the survival rate of the HF10 group was significantly higher than that of the erlotinib group, but there was no significant difference in survival between the use of HF10 alone and the combination therapy, which shows that the clinical application of this combination therapy is yet to be investigated.

The most common and deadly form of pancreatic cancer is pancreatic ductal adenocarcinoma (PADC), which is strongly associated with diabetes. Some studies have shown that long-standing type 2 diabetes is a risk factor for PADC [37]. Epidemiological studies have shown that metformin has immunomodulatory and antitumour effects, and the use of metformin has improved the survival of diabetic pancreatic adenocarcinoma patients [38]. Mohamed Abdelmoneim et al. first investigated the anti-pancreatic cancer effects of metformin in combination with HF10 [39]. The results of in vivo experiments in a low-immunogenic Pan02 mouse model of PDAC showed that combined treatment with HF10 produced significant anti-pancreatic cancer effects by enhancing the high infiltration of CD8 TIL and significantly prolonged survival in mice, but the in vitro experiments did not show any significant cytotoxic effects. This study may provide additional insights for future studies on the combination of oHSV and metformin for the treatment of pancreatic cancer.

## Tumours of the reproductive system

### Breast *cancer*

The latest global cancer burden data for 2020 released by the World Health Organisation’s International Agency for Research on Cancer (IARC) shows that the incidence and mortality of breast cancer is increasing year by year [40]. Although there are a variety of treatment options for breast cancer, none of them are without limitations. Currently, paclitaxel-containing therapy is the main chemotherapy option for breast cancer, the toxicity and side effects of the drug are the key factors limiting its widespread use. Zeng Weigen and his team investigated the combination of G47Δ and paclitaxel for the treatment of breast cancer [41]. They used human breast cancer cell lines MCF-7 and MDA-MB-468 in a combined cytotoxicity assay, and found that the combination of G47Δ and paclitaxel had a synergistic effect on breast cancer, and the cytotoxicity was dose-dependent. In order to investigate the interaction between the two, further experiments revealed that the replication of G47Δ was not affected by paclitaxel, and G47Δ significantly enhanced the ability of paclitaxel to induce apoptosis in tumour cells by inhibiting mitosis. In addition to this, they also did in vivo experiments and found that this combination therapy not only showed synergistic effects in the in vivo experiments, but also significantly reduced the weight of the tumour and most importantly did not show additional complications.

Temozolomide (TMZ) is an oral alkylating agent that crosses the blood–brain barrier and is primarily used to treat malignant gliomas. Jingjing Fan and her research team investigated the effects of the combination of G47Δ and TMZ in the treatment of breast cancer in vivo and in vitro [42]. The results showed that G47Δ and TMZ synergistically inhibited cancer cell viability, induced cancer cell cycle arrest, promoted apoptosis, and up-regulated the expression of DNA-associated damage genes in cancer cells, which could be of potential clinical value in the treatment of breast cancer. However, the experiment still has some limitations and more in-depth studies are needed.

Bevacizumab was challenged for use in the treatment of advanced breast cancer more than a decade ago and even received fast-track approval from the FDA in 2008. Tangevin and his researchers studied and evaluated the effects of HF10 in combination with bevacizumab in an experimental model of human breast cancer xenografts [43]. The results showed that bevacizumab enhanced viral distribution within tumour cells, increased the number of apoptotic cells and induced synergistic anti-tumour effects. One of the other interesting findings was that bevacizumab reduced angiogenesis compared to controls, whereas HF10-induced neoangiogenesis was significantly higher. However, the combination therapy clearly inhibited neoangiogenesis compared with HF10 treatment alone. It can be seen that the combination therapy of HF10 and bevacizumab is worthy of further study in the treatment of human breast cancer.

### Cervical cancer

Cervical cancer is one of the most common gynaecological cancers in women. Persistent high-risk human papillomavirus (HPV) infection is the main risk factor for cervical cancer and in recent years, HPV vaccine has achieved good results in the prevention of this type of cervical cancer. In addition to immunotherapy, it has also become a new option for cervical cancer treatment. Currently the anti-tumour efficacy of ICIs is a research hotspot, of which the main ones are PD-1 and PD-L1. oHSV specific anti-tumour ability combined with ICIs might be a better therapeutic option. In 2021 Masahiro Kagabu and his researchers demonstrated the cytotoxic efficacy of triple-mutated oncolytic herpes virus (T-01) to inhibit the growth of cervical cancer cells of HPV-associated phenotypes [44]. Subsequently in 2023 Masahiro Kagabu published the first report on the use of a bilateral tumour model combining oHSVT-01 and ICIs for the treatment of cervical cancer [45]. The results of the study reported that the combination of T-01 and PD-1Ab significantly increased the number of tumour-specific T cells in the tumour group, but the combination therapy did not show a significant anti-tumour effect compared to T-01 monotherapy, so concomitant administration of the two in the treatment of HPV-associated cervical cancer needs to be carefully considered.

### Prostate cancer

Prostate cancer is a more common cancer in older men, with the incidence rate gradually increasing with age, and most of the patients are already in the middle or late stage when they are found. Current mainstream treatments are basically based on monotherapy. The development of prostate cancer is dependent on the action of androgens, and endocrine therapy is used to suppress the action of androgens to achieve the therapeutic effect. Hiroshi Fukuhara et al. studied G47Δ in combination with Androgen Ablation for the treatment of human prostate cancer [46]. In mouse syngeneic and human xenograft models of prostate cancer, this combination therapy not only rapidly inhibited tumour growth but also prolonged the survival of mice compared to either therapy alone.

Paclitaxel is a classic oncological chemotherapeutic agent, and BJ Passer and colleagues investigated the combination of G47Δ with paclitaxel in prostate cancer [47]. Experimental data observed a synergistic killing of prostate cancer cells with this combination therapy, G47Δ acts on mitotically blocked cells to enhance this effect. Subsequently in 2013 BJ Passer team published an article on the combination of vinblastine (VB) and oHSV in prostate cancer. VB is a microtubule disrupting agent (MDA) that inhibits mitosis to prevent cancer cells from growing and dividing [48]. Six MDAs were tested and it was found that VB was effective in killing not only prostate cancer cells but also all endothelial cells. Combination therapy of "armed" oncolytic oHSV (NV1042) with VB has significant anti-angiogenic and anti-tumour effects and is expected to overcome the resistance to tumours arising from anti-angiogenic monotherapy.

Diane Ojo et al. identified prostate cancer stem-like cells (PCSCs) in mouse prostate cancer by single-cell RNA sequencing analysis of human and mouse prostate block and indicated that such cells play an important role in maintaining the stability of prostate tissues and recovery after injury [49]. The PIK3 signalling pathway is essential for the maintenance of PCSCs, and Wang Lei et al. used the PI3K inhibitor BKM120 to study its combined effect with oHSV in prostate cancer [50]. The experimental results found that oHSV and BKM120 had a synergistic killing effect on PCSC in vitro and significantly inhibited tumour growth. In the face of prostate cancer treatment, we should no longer be limited to a single treatment option, and exploring more combination treatments may be a better direction for research.

## Tumours of other types or systems

### NuT carcinoma

NuT carcinoma (NC) is a rare and highly aggressive malignant tumour of unknown tissue origin, which is prevalent in the midline organs of children and young adults. Current treatments include surgery, radiotherapy and chemotherapy, but the median survival is only 6.7 months and the prognosis is very poor [51]. T-VEC, a modified HSV-1-based lysosomal virus therapy, was the first FDA-approved lysosomal virus therapy and is now a widely recognised lysosomal virus product. Paul and his researchers first studied and demonstrated that T-VEC could effectively infect and replicate in NC cell lines, showing strong cytotoxic effects [52]. Six NC cell lines containing BRD4-NUT fusion proteins were used in the experiments, and all six NC cell lines were treated with either iBET compounds or single agents to determine their anti-tumour effects in NC treatment. Between the anti-NC effects of both approaches, further experimental results showed that iBET treatment after T-VEC infection enhanced its cytotoxic effects and that iBET treatment did not impair viral replication.

## Conclusion

In this paper, we have introduced the progress of oHSV combination therapy for anti-tumour treatment, mainly describing the progress of oHSV combination therapy in tumours of the nervous system, the digestive system, the reproductive system, as well as some refractory tumours in other systems, and it can be seen that the combination therapy of oHSV in a variety of tumours has achieved satisfactory results. However, there are some problems and challenges in many aspects, such as the mechanism of some combination therapies is still unclear; some experiments are only limited to in vivo experiments, which is obviously insufficient; some combination therapies do not show more significant anti-tumour effects than monotherapy, etc., which need and deserve further in-depth research. In addition, when the effect of monotherapy is not obvious in other systemic tumours, can we also research in the direction of oHSV combination therapy, which may have different gains. It is evident that future exploration and research are necessary to seek more and better oHSV combination therapy to improve the anti-tumour effect and open up more new roads for tumour treatment.

## Data Availability

The datasets generated during and analyzed during the current study are publicly available. No datasets were generated or analysed during the current study.
